# Feasibility of Mechanical Extrusion to Coat Nanoparticles with Extracellular Vesicle Membranes

**DOI:** 10.3390/cells9081797

**Published:** 2020-07-29

**Authors:** Jan Van Deun, Quentin Roux, Sarah Deville, Thibaut Van Acker, Pekka Rappu, Ilkka Miinalainen, Jyrki Heino, Frank Vanhaecke, Bruno G. De Geest, Olivier De Wever, An Hendrix

**Affiliations:** 1 Laboratory of Experimental Cancer Research, Department of Human Structure and Repair, Ghent University, 9000 Ghent, Belgium; Jan.VanDeun@uk-erlangen.de (J.V.D.); Quentin.Roux@UGent.be (Q.R.); Sarah.Deville@UGent.be (S.D.); olivier.dewever@ugent.be (O.D.W.); 2 Cancer Research Institute Ghent, 9000 Ghent, Belgium; 3 Department of Analytical Chemistry, Ghent University, 9000 Ghent, Belgium; Thibaut.VanAcker@UGent.be (T.V.A.); Frank.Vanhaecke@UGent.be (F.V.); 4 Department of Biochemistry, University of Turku, 20500 Turku, Finland; pekrappu@utu.fi (P.R.); jyheino@utu.fi (J.H.); 5 Biocenter Oulu, Department of Pathology, Oulu University Hospital, University of Oulu, 90220 Oulu, Finland; ilkka.miinalainen@oulu.fi; 6 Department of Pharmaceutics, Ghent University, 9000 Ghent, Belgium; Br.DeGeest@UGent.be

**Keywords:** extracellular vesicle, exosome, microvesicle, biomimetics, gold nanoparticle, functionalization, drug delivery, cancer

## Abstract

Biomimetic functionalization to confer stealth and targeting properties to nanoparticles is a field of intense study. Extracellular vesicles (EV), sub-micron delivery vehicles for intercellular communication, have unique characteristics for drug delivery. We investigated the top-down functionalization of gold nanoparticles with extracellular vesicle membranes, including both lipids and associated membrane proteins, through mechanical extrusion. EV surface-exposed membrane proteins were confirmed to help avoid unwanted elimination by macrophages, while improving autologous uptake. EV membrane morphology, protein composition and orientation were found to be unaffected by mechanical extrusion. We implemented complementary EV characterization methods, including transmission- and immune-electron microscopy, and nanoparticle tracking analysis, to verify membrane coating, size and zeta potential of the EV membrane-cloaked nanoparticles. While successful EV membrane coating of the gold nanoparticles resulted in lower macrophage uptake, low yield was found to be a significant downside of the extrusion approach. Our data incentivize more research to leverage EV membrane biomimicking as a unique drug delivery approach in the near future.

## 1. Introduction

Nanoparticles (NP) are increasingly considered as ideal drug carriers for cancer therapy because of improved solubility, in vivo stability, distribution, targeting options, controlled drug release and decreased therapy resistance [1]. The current standard for stealth in nanoparticle drug delivery is poly (ethylene glycol) (PEG) coating to improve circulation time by decreasing clearance by the reticuloendothelial system (RES) [2]. Notable downsides of this approach include potential immunological response and absence of active targeting [3]. Indeed, PEGylated nanoparticles rely on passive targeting to primary tumors via the enhanced permeability and retention (EPR) effect caused by leaky vasculature. However, whether the EPR effect has any physiological relevance in human subjects is under increased scrutiny [4].

Bottom-up conjugation of nanoparticles with multiple targeting ligands and adhesion molecules to mimic biological entities is problematic for technical reasons such as the denaturation of proteins during the conjugation process and the overall difficulty of duplicating biological complexity on a nanoscale [5]. To overcome these issues, functionalization of nanoparticles with biological membranes has been suggested [6]. Red blood cells, platelets, neutrophils, leukocytes, cancer cells and bacteria have successfully been used as a membrane source for their biological compatibility, retention of cellular properties and increase in nanoparticle circulation time [5,7,8,9,10,11]. Nevertheless, these membranes are not expected to be enriched in surface proteins that are directly involved in tumor cell communication.

Research into extracellular vesicles (EV) has yielded important biological insights and raised the prospect of developing novel therapeutics [12]. EV are nanometer-sized membrane vesicles that are released by many cell types, contain lipids, proteins, nucleotides and metabolites, and can travel to distant tissues to influence various physiological and pathological functions [13,14]. Tumors consist of heterogeneous clonal populations that can share and exchange EV with local and distant ecosystems (e.g., metastasis) [15]. The discovery that EV make up a natural mechanism for information transfer between cells has stimulated interest into their potential use as a new drug delivery platform [16,17]. The intraluminal EV cargo is naturally protected from degradation in the circulation. The anti-phagocytic membrane protein CD47, which reduces uptake of cancer cells by monocytes and macrophages is also present on EV [18,19]. EV possess intrinsic cell targeting properties that allow cell type-specific interactions [20]. Finally, EV are nearly non-immunogenic when used autologously. Several clinical trials have demonstrated the safety of EV administration in humans [21]. EV have been modified by manipulating their parent cells; either through genetic or metabolic engineering, or by introducing exogenous material that is subsequently incorporated into secreted EV [22,23,24]. Alternatively, EV have been directly functionalized using strategies such as hydrophobic insertion, covalent surface chemistry and membrane permeabilization [16,25,26].

**Figure 1:**
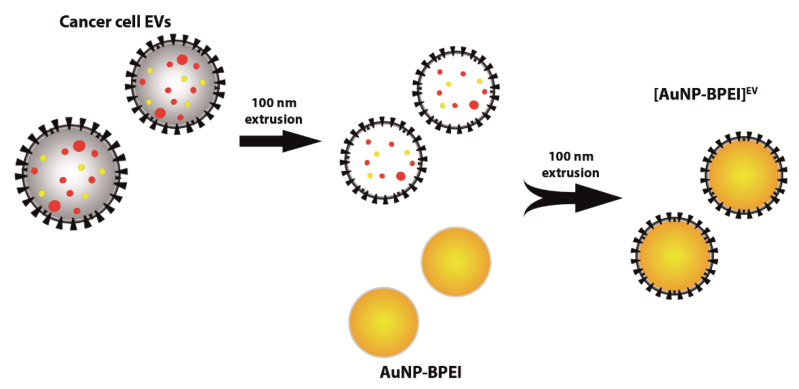
Functionalization of AuNP with cancer EV membranes. Density gradient-purified cancer cell EV are first extruded through a 100 nm membrane, resulting in size homogenization and alteration of EV content, but retention of membrane composition and orientation. Extruded EV are then mixed with AuNP-BPEI and extruded a second time, resulting in the formation of EV membrane-cloaked AuNP.

In this study, we provide proof-of-concept for conferring potential immune-evading and specific targeting properties of cancer EV upon nanoparticles by coating them with intact EV membranes. We show that the uptake of cancer EV is affected by the presence of intact EV membrane surface proteins, which include CD47. Utilizing a top-down approach, 70 nm positively-charged gold nanoparticles (AuNP-BPEI) were coated with 4T1 mouse mammary cancer EV membranes by means of mechanical extrusion (Figure 1). We demonstrate that AuNP are successfully functionalized with breast cancer EV membranes, which results in decreased uptake by macrophages.

## 2. Materials and Methods

### 2.1. Reagents

The following primary antibodies were used for immunostaining: rat monoclonal CD9 (1:1000) (KMC8, BD Biosciences, Erembodegem, Belgium), rat monoclonal CD47 (1:250) (MIAP301, BD), mouse monoclonal anti-Alix (1:1000) (2171, Cell Signaling, Danvers, MA, USA), mouse monoclonal TSG101 (1:1000) (sc-7964, Santa Cruz, Santa Cruz, CA, USA), mouse monoclonal anti-flotillin 1 (1:1000) (18/Flotillin-1, BD). Branched polyethylene imine (BPEI)-coated gold nanoparticles (AuNP-BPEI) with a diameter of 70 nm were purchased from nanoComposix. PKH67 fluorescent cell linker kit was acquired from Sigma (Overijse, Belgium).

### 2.2. Cell Culture

The murine triple-negative mammary cancer and macrophage cell lines 4T1_luc (Sibtech, Brookfield, CT, USA) and J774A1 (Sigma) were maintained in Dulbecco’s minimal essential medium (DMEM) (Invitrogen, Merelbeke, Belgium) supplemented with 10% fetal bovine serum, 100 U/mL penicillin and 100 mg/mL streptomycin, and incubated at 37 °C, 5% CO_2_. Cell cultures were regularly tested and found to be negative for mycoplasma contamination using the MycoAlert Mycoplasma Detection Kit (Lonza, Bornem, Belgium) [27].

### 2.3. Preparation of Conditioned Medium

Cell cultures in T175 flasks were washed three times using DMEM followed by 24 h incubation with 15 mL EV-harvesting medium at 37 °C and 5% CO_2_. EV-harvesting medium was DMEM supplemented with 0.5% EV-depleted fetal bovine serum (EDS). EDS was obtained through 18 h centrifugation of fetal bovine serum at 100,000× *g* and subsequent filtering through a 0.2 µm Whatman filter Conditioned medium (CM) was harvested and centrifuged for 10 min at 200 g and 4 °C to remove detached cells, followed by a 0.45 µm cellulose acetate filtration (Corning, Amsterdam, The Netherlands) to remove larger particles. Next, CM was concentrated approximately 200 times to 1 mL using a Centricon Plus-70 centrifugal filter device with a 10 K nominal molecular weight limit regenerated cellulose filter (Merck Millipore, Burlington, MA, USA).

### 2.4. EV Separation

A discontinuous iodixanol gradient was used as described previously [28]. Solutions of 5, 10, 20 and 40% iodixanol were made by mixing appropriate amounts of a homogenization buffer (0.25 M sucrose, 1 mM EDTA, 10 mM Tris-HCL, (pH 7.4)) and an iodixanol working solution. This working solution was prepared by combining a working solution buffer (0.25 M sucrose, 6 mM EDTA, 60 mM Tris-HCl, pH 7.4) and a stock solution of OptiPrep^TM^ (60% (*w*/*v*) aqueous iodixanol solution, Axis-Shield, Oslo, Norway). The gradient was formed by layering 4 mL of 40%, 4 mL of 20%, 4 mL of 10% and 3.5 mL of 5% solutions on top of each other in a 16.8 mL open top polyallomer tube (Beckman Coulter, Suarlee, Belgium). One milliliter of concentrated CM sample was overlaid onto the top of the gradient, which was then centrifuged for 18 h at 100,000× *g* and 4 °C (SW 32.1 Ti rotor, Beckman Coulter). Gradient fractions of 1 mL were collected from the top of the gradient, with fractions 9 and 10 being pooled (corresponding to a density of 1.087–1.109 g/mL) and used for subsequent size-exclusion chromatography (SEC) separation of EV from the iodixanol polymer [29]. To prepare the SEC column, Sepharose CL-2B (GE Healthcare, Machelen, Belgium) was washed three times with PBS. A nylon net with 20 µm pore size (NY2002500, Merck Millipore) was placed on the bottom of a 10 mL syringe (BD Biosciences), followed by stacking of 10 mL washed Sepharose. A 2 mL sample was loaded on top of the SEC column and fractions of 1 mL eluate were collected. EV-containing fractions (4–7) were pooled and concentrated to 100 µL using Amicon Ultra-2 10K filters (Merck Millipore), and stored at −80 °C.

For PKH labelling, the top-down density gradient was replaced by a bottom-up gradient. The 2 mL sample was mixed with a 50% iodixanol solution to form the 40% layer of the gradient, followed by layering the other fractions and adding 1 mL of PBS on top. The rest of the procedure was completed as with the top-down gradient.

### 2.5. Proteinase K Treatment

EV suspensions (concentration of E10 particles µL^−1^) were treated with a final concentration of 2 µg/mL Proteinase K (PK) for 30 min at 37 °C under gentle agitation. PK treatment was inhibited by addition of PMSF at a final concentration of 5 mM for 10 min at room temperature under gentle agitation. PK-treated EV (PK-EV) were purified from residual PK and PMSF by diluting with PBSD- to a volume of 2 mL and performing SEC as described above.

### 2.6. Extrusion

To prepare a homogenous EV population, a 600 µL water suspension of 1E11 4T1-luc EV was extruded 5 times through a 100 nm polycarbonate porous membrane using an Avanti mini extruder. After extrusion, the sample was mixed with 3E10 AuNP-BPEI before being extruded 15 times as before. An excess of extruded 4T1 EV was used to maximize the EV membrane-AuNP interaction during extrusion. The resulting EV membrane-cloaked AuNP ([AuNP-BPEI]^EV^) were centrifuged for 10 min at 800× *g* to remove excess EV material.

### 2.7. PKH67 Labelling

EV (whether or not treated with proteinase K) were suspended in a buffer with 0.2% BSA and PKH67 solution (ca. 10 µL in 100 µL diluent C per 10E11 EV) was added. After 5 min incubation at room temperature, the reaction was terminated by adding 100 µL 10% EDS DMEM. To separate free dye and dye micelles from labelled EV, a bottom-up density gradient and SEC were performed as described above. It was observed that dye micelles end up in fractions 12–13 of the gradient (Figure S1).

### 2.8. Western Blotting

Equal particle numbers were suspended in reducing sample buffer (0.5 M Tris-HCl (pH 6.8), 43% glycerol, 9.2% SDS, 5% 2-mercaptoethanol, 5% bromophenol blue) and boiled for 5 min at 95 °C. Proteins were separated by SDS-PAGE (SDS polyacrylamide gel electrophoresis), transferred to nitrocellulose membranes, blocked in 5% nonfat milk in PBS with 0.5% Tween-20, and immunostained using the primary antibodies described in the reagents section. Blots were developed using the WesternBright Sirius reagent and visualized on a Proxima AQ-4 system (Isogen Life Science, Temse, Belgium). For quantification, non-saturated bands were selected and analyzed using Image J.

### 2.9. Nanoparticle Tracking Analysis

Nanoparticle tracking analysis (NTA) was performed using a NanoSight LM10-HS microscope (Malvern, Amelo, The Netherlands) equipped with a 405 nm laser. Three or four 30 s videos were recorded of each sample with camera level 13 for EV and 5 for AuNP. After each video, the sample was advanced through the chamber to avoid repeated measurement of identical particles in the field of view. Videos recorded for each sample were analyzed with NTA software version 3.2 with detection threshold kept constant at 3. Samples were diluted with molecular water until particle concentration was within the linear concentration range of the NTA software (3 × 10^8^–1 × 10^9^ particles/mL).

### 2.10. Electron Microscopy

EV, AuNP or [AuNP-BPEI]^EV^ were deposited on Formvar carbon coated, glow-discharged grids. After 20 min, the grids were incubated in a blocking serum containing 1% BSA in PBS. Antibodies and gold conjugates were diluted in 1% BSA in PBS. In case of immunostaining, the grids were exposed to the primary rat anti-mouse CD9 antibody (KMC8, 10 mg/mL) for 20 min, followed by goat anti-rat secondary antibody (Zymed, San Francisco, CA, USA) for 20 min and protein A-gold complex (10 nm size) (CMC Utrecht, Utrecht, Netherlands) for 20 min. The blocking efficiency was controlled by performing the labelling procedure in the absence of primary antibody. The grids were stained with neutral uranylacetate and embedded in methylcellulose/uranyl acetate and examined in a Tecnai Spirit transmission electron microscope (Thermo Fisher Scientific FEI, Merelbeke, Belgium). Images were captured by Quemesa charge-coupled device camera.

### 2.11. Zeta Potential

The Zeta potential of particles was measured on a Zetasizer Nano-ZS instrument (Malvern). Three measurements per sample were taken, with samples suspended in water at room temperature.

### 2.12. Mass Spectrometry

EV and extruded EV suspensions containing an equal total number of particles (1.3E11, as measured by NTA) were processed using filter-aided sample preparation. EV suspensions were mixed with SDT-lysis buffer (2% SDS, 500 mM Tris/HCl pH 7.6, 0.5 M DTT) at a 4:1 sample-to-buffer ratio and incubated for 5 min at 95 °C. After clarification of the lysate by centrifugation (16,000× *g*, 5 min), samples were mixed with 300 µL UA buffer (8 M urea in 0.1 M Tris/HCl pH 8) on top of a Microcon 10 kDa centrifugal filter unit (Merck Millipore). Filter units were centrifuged twice for 40 min at 14,000× *g* with addition of 200 µL UA buffer in between. One hundred microliters of IAA solution (0.05 M iodoacetamide in UA buffer) was then added and incubated at room temperature for 30 min. Filter units were centrifuged thrice for 30 min at 14,000× *g* with addition of 100 µL UA buffer in between. One hundred microliters of DB buffer (1 M urea in 0.1 M Tris/HCl pH 8.5) was then added followed by centrifugation for 30 min at 14,000× *g*. This step was repeated once. Filter units were then transferred to new collection tubes, followed by addition of 40 µL Lys-C/Trypsin (0.1 µg/µL, dissolved in DB buffer). After overnight incubation at 37 °C, 100 µL DB buffer was added and filters centrifuged for 15 min at 14,000× *g*. This step was repeated once. Collected peptides were desalted, dried, dissolved in 1% formic acid and loaded on a nanoflow HPLC system (Easy-nLCII, Thermo Fisher Scientific) coupled to the Q Exactive mass spectrometer (Thermo Fisher Scientific) equipped with a nano-electrospray ionization source. The tandem mass spectra were analyzed by MaxQuant software [30] version 1.5.2.8 using mouse unreviewed and reviewed sequences with isoforms of UniProt release 2018_01. Carbamidomethyl (C) and oxidation (M) were used as fixed and variable modifications, respectively.

### 2.13. EV Uptake Analysis by Flow Cytometry

Cells were seeded in a 6-well plate at a density of 300,000 cells/well (J774A1) or 200,000 cells/well (4T1). After 24 h, cells were incubated with 4E09 PKH67 membrane-labelled EV or PK-EV.

Negative and positive controls for flow cytometry were obtained by applying the PKH67 labelling protocol on a suspension of PKH67 without EV, starting with the same volume of PKH67 stock solution that was used for EV labelling. As negative control, cells were incubated with the same volume of PKH67-only suspension (after bottom-up density gradient, collecting F9–10, SEC and ultrafiltration to 100 µL) as the volume required to obtain 4E09 EV (i.e., 8 µL). This sample is referred to in the text as ‘Control’. As positive control, PKH67 micelle-containing F12–13 (corresponding to a density of 1.156–1.201 g/mL) of the bottom-up density gradient were collected and processed by SEC and ultrafiltration. The final suspension was adjusted to the same fluorescence as the labelled EV by measuring on a fluorescence plate reader, and the same volume was added to cells. This sample is referred to in the text as ‘PKH67 micelles’.

After 16 h of incubation, cells were washed with 1 mL PBSD^+^ and 1 mL Moscona buffer, and then detached by scraping in 1 mL PBSD^−^ (J774A1) or trypsinization (4T1). Detached cells were washed once in PBS and final pellets dissolved in 500 µl PBSD^-^. Cell suspensions were then measured on a BD Acurri C6 flow cytometer. One hundred thousand events were collected per sample and gated using control cells that were not incubated with any EV or AuNP. Data were analyzed using FlowJo software and statistical analysis was done with GraphPad Prism.

### 2.14. Confocal Microscopy

Cells were seeded in 12 mm diameter confocal dishes (Thermo Fisher Scientific) at 45,000 cells/dish. After 24 h, 6E08 PKH67 membrane-labelled EV or PK-EV were added. After 16 h of incubation, cell membranes were stained with a cholera toxin subunit B-Alexa Fluor 555 conjugate (Sigma) and cells were imaged on a Leica DMI6000 confocal microscope coupled to an Andor DSD2 scanner and a Zyla5.5 CMOS camera. Images were processed with ImageJ.

### 2.15. Luminex Assay

Concomitantly with cell collection for flow cytometry, J774A1 macrophage-conditioned medium was collected after 16 h incubation with EV or PK-EV. Collected medium was centrifuged for 10 min at 200 g to remove cells. An aliquot of 200 µL of the supernatant was frozen at −80 °C. Analysis of 44 cytokines and chemokines was carried out by Eve Technologies (Calgary, Canada) using a Luminex bead-based assay (Mouse Cytokine Array/Chemokine Array 44-Plex). Statistical analysis of results was done with GraphPad Prism.

### 2.16. AuNP Uptake by Inductively Coupled Plasma-Mass Spectrometry (ICP-MS)

J774A1 cells were seeded in a 6-well plate at a density of 300,000 cells/well. After 24 h, cells were incubated with 9E08 PKH67 AuNP-BPEI or [AuNP-BPEI]^EV^. After 16 h of incubation, cells were washed with 1 mL PBSD^+^ and 1 mL Moscona buffer and detached by scraping in 1 mL PBSD^-^. Detached cells were washed once in PBSD^-^ and final pellets were dissolved in 500 µL PBSD^−^. In order to degrade the organic matrix and dissolve the AuNP prior to analysis, aliquots of 300 µL were digested on a hot plate at 100 °C for 24 h in closed Teflon Savillex beakers using 2.7 mL of an acid mixture (3:1) of sub-boiled pro-analysis grade 12 M HCl (ChemLab, Zedelgem, Belgium) and 14 M HNO_3_ (ChemLab). After the acidic digestion step, aliquots of 250 µL were transferred to metal-free 15 mL centrifuge tubes (VWR International) and 100 µL of a 100 µg/L Ir solution (Inorganic Ventures, Christiansburg, VA, USA) was added as an internal standard. The solutions were diluted to a final volume of 10 mL with ultra-pure water, purified by a Milli-Q Element water purification system (Millipore, France). For Au quantification, external calibration standards were prepared with different Au concentrations (0, 0.1, 0.5, 1, 2.5, 5 and 10 µg/L Au in 2.25% aqua regia) and an Ir internal standard concentration of 1 µg/L Ir. Determination of the Au concentration was performed using a quadrupole-based XSeries-II ICP-MS unit (Thermo Scientific).

### 2.17. EV-TRACK

We have submitted all relevant data of our experiments to the EV-TRACK knowledgebase (EV-TRACK ID: EV180012) [31].

## 3. Results

### 3.1. EV Membrane Proteins Differentially Affect Uptake Depending on Cell Type

**Figure 2:**
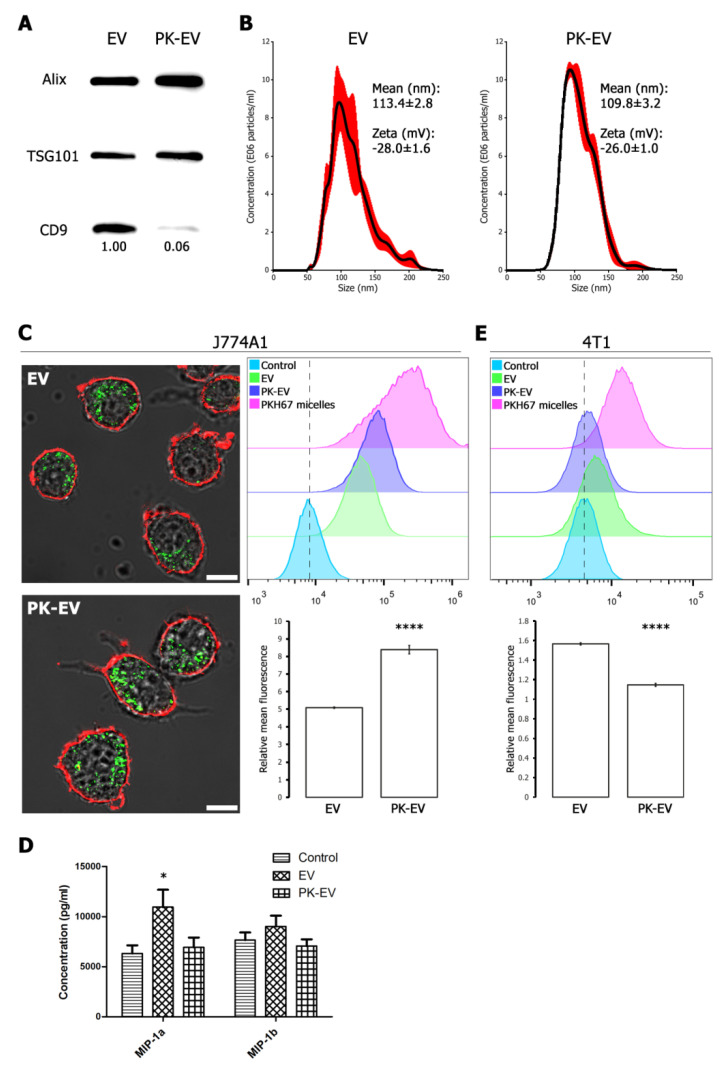
Presence of EV surface membrane proteins affects uptake by cells. (**A**) Western blot for intraluminal markers Alix and TSG101, and transmembrane protein CD9 in EV versus PK-EV (equal particle numbers loaded). Decrease in CD9 signal was quantified using Image J. (**B**) NTA size distribution of EV versus PK-EV, depicted as mean (black line) with standard error (red shaded area). Inset: mean size ± standard error; mean zeta potential ± standard error. (**C**) Left panels: representative confocal images of J774A1 macrophages after 16 h incubation with EV or PK-EV. Red = Cholera toxin-B Alexa Fluor 555 conjugate membrane staining; Green = PKH67 membrane-labelled (PK-)EV. Scale bar = 10 µm. Right panels: EV and PK-EV uptake by J774A1 macrophages quantified by flow cytometry with appropriate negative and positive controls (see Methods section). Dotted line indicates mean fluorescence of control. Relative mean fluorescence of cells treated with EV versus PK-EV is shown with standard deviation. Asterisks indicate significantly different values (****, two-sided *t*-test, *p* = 0.00002). (**D**) Concentration of cytokines MIP-1a and MIP-1b in supernatant of J774A1 macrophages after 16 h stimulation with control medium versus medium containing EV or PK-EV. Mean concentration is shown with standard error. Asterisk indicates significantly different values (*, two-sided *t*-test, *p* = 0.03). (**E**) EV and PK-EV uptake by 4T1 cancer cells quantified by flow cytometry with appropriate negative and positive controls (see Methods section). Dotted line indicates mean fluorescence of control. Relative mean fluorescence of cells treated with EV versus PK-EV is shown with standard deviation. Asterisks indicate significantly different values (****, two-sided *t*-test, *p* = 0.000003).

To evaluate the potential of cancer cell-derived EV membranes to functionalize nanoparticles, we investigated the influence of breast cancer EV membrane surface proteins on uptake by cells. EV were treated with 2 µg/mL proteinase K (PK) to shave membrane surface proteins (PK-EV). This approach was validated via Western blot for a selection of intraluminal and surface membrane marker proteins (Figure 2A and Figure S1). Intraluminal EV markers Alix and TSG101 remained stable during PK treatment, while an antibody directed against the surface-exposed part of CD9 showed that this epitope was largely removed. Quantification of the Western blot signal of CD9 versus Alix and TSG101 revealed a removal efficiency of 94%. Nanoparticle tracking analysis (NTA) and zeta analysis revealed no significant differences in size distribution or zeta potential of EV versus PK-EV (Figure 2B). EV and PK-EV were then fluorescently labelled with PKH67 lipid dye, a procedure involving a bottom-up density gradient to robustly separate labelled EV from dye micelles (Figure S2). J774A1 macrophages were incubated for 16 h with EV or PK-EV and their uptake was confirmed by confocal microscopy (Figure 2C, left panels). Quantification via flow cytometry revealed significantly higher uptake of PK-EV (65% increase in mean fluorescence signal of PK-EV versus EV), indicating an inhibiting role of membrane surface proteins in macrophage uptake (Figure 2C, right panels). To verify the effect of EV versus PK-EV uptake on the macrophages, a Luminex assay was performed for 44 cytokines, of which 17 could be detected in the macrophage conditioned medium (Figure S3). Despite lower uptake, macrophages were stimulated more by native EV than PK-EV as demonstrated by a higher secretion of macrophage inflammatory protein (MIP)-1a and MIP-1b (Figure 2D). Interestingly, an opposite effect of PK treatment was seen on autologous EV uptake by the 4T1 cells (Figure 2E). Even though overall uptake was relatively small, PK-EV were significantly less taken up than EV (27% decrease in mean fluorescence signal of PK-EV versus EV). Overall, these results demonstrate that functionalization of nanoparticles with cancer cell EV membranes could result in specific targeting to cancer cells while reducing non-specific uptake by macrophages.

### 3.2. Cancer EV Retain Membrane Protein Orientation and Composition after Extrusion

**Figure 3:**
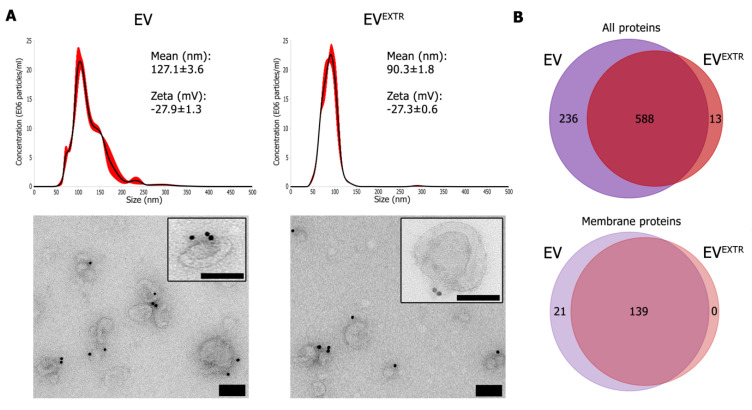
Mechanical extrusion does not significantly alter EV physical and biochemical characteristics. (**A**) Upper panels: NTA size distribution of EV versus extruded EV, depicted as mean (black line) with standard error (red shaded area). Inset: mean size ± standard error; mean zeta potential ± standard error. Lower panels: CD9 immuno-electron microscopy images of EV versus extruded EV. Scale bars: 100 nm. (**B**) Venn diagrams of proteins identified by mass spectrometry in EV versus extruded EV, including either all proteins (upper diagram) or proteins annotated as membrane proteins (lower diagram).

To evaluate feasibility of the extrusion approach, we investigated whether EV would maintain their characteristics after extrusion through a 100 nm membrane. Size-exclusion chromatography was used to separate extruded EV (EV^EXTR^) from EV luminal content released during the extrusion process. As expected, EV^EXTR^ had a more homogeneous size distribution as shown by NTA (Figure 3A, top panels). The concentration of EV was similar before and after extrusion (1.17 × 10^9^ ± 1.66 × 10^8^ before vs. 9.62 × 10^8^ ± 1.07 × 10^8^ after (mean particles per mL ± standard error)). Zeta potential also remained the same, suggesting similar surface composition and orientation of the EV membrane. Immuno-electron microscopy confirmed the smaller size after extrusion and did not reveal any adverse effects on membrane composition and orientation, as indicated by membrane surface localization of CD9 (Figure 3A, bottom panels). The protein profiles of native EV versus EV^EXTR^ were determined by mass spectrometry (Figure 3B).

In total, 837 proteins were detected in the samples, of which 588 (70%) were shared between both. Thirteen proteins (2%) were detected only in EV^EXTR^ and 236 (23%) only in native EV. Focusing on the 160 proteins that are annotated as membrane proteins [32], the large majority of these (139 proteins: 87%) are shared between native and EV^EXTR^. These proteins include several tetraspanins (CD9, CD63), integrins (ITGA2, ITGA3, ITGA5, ITGA6, ITGA8, ITGAV, ITGB1, ITGB4, ITGB5, ITGB6) and adhesion molecules (EPCAM, ALCAM, CD44). Interestingly, the anti-phagocytic marker CD47 was detected in 4T1 EV and EV^EXTR^. Collectively, these results indicate that extrusion through a 100-nm membrane does not alter 4T1 EV membrane protein composition or orientation, and could be suitable as a method for functionalizing nanoparticles with EV membranes.

### 3.3. Gold Nanoparticle Functionalization with EV Membranes

**Figure 4:**
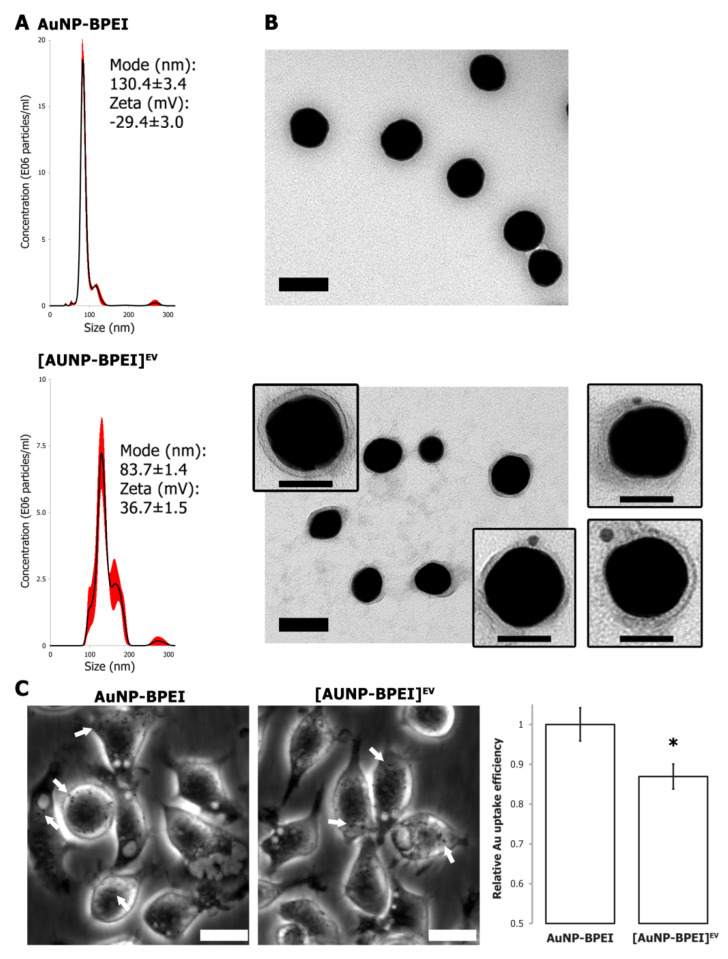
Functionalization of AuNP by EV membranes. (**A**) NTA size distribution of AuNP-BPEI versus AuNP-BPEI cloaked with EV membranes, depicted as mean (black line) with standard error (red shaded area). Inset: size mode (nm) and mean zeta potential (mV) ± standard error. (**B**) Transmission electron microscopy of AuNP-BPEI and EV-cloaked AuNP-BPEI. Scale bar: 100 nm. Inserts: CD9 immunoelectron microscopy of EV-cloaked AuNP-BPEI. Scale bar: 50 nm. (**C**) Left panels: Phase contrast images of J774A1 macrophages after 16 h incubation with AuNP-BPEI or [AuNP-BPEI]^EV^. White arrows indicate phagocytosed AuNP aggregates. Scale bar: 20 µm. Right panel: Quantification of AuNP uptake by determination of total Au concentration via ICP-MS. Asterisk indicates significantly different value (*, two-sided t-test, *p* = 0.01).

To achieve successful coating of AuNP with EV membranes, 70 nm sized AuNP with a branched polyethylene imine (BPEI) coating were used. AuNP-BPEI were characterized by NTA, zeta potential measurement and EM (Figure 4A).

The positive surface charge maximizes interactions when mixing AuNP-BPEI with negatively charged EV (Figure S4). Extruding the mixture 15 times through a 100 nm membrane resulted in an increase in size and a shift in zeta potential from positive to negative (Figure 4A). Visualization by EM clearly demonstrated a membrane that was tightly fitted around the AuNP-BPEI after extrusion, thus revealing the creation of bona fide EV membrane-cloaked AuNP-BPEI or [AuNP-BPEI]^EV^ (Figure 4B). Even though limited aggregation was observed with NTA, [AuNP-BPEI]^EV^ showed higher stability than AuNP-BPEI when suspended in serum-containing medium (Figure S5). Importantly, immune-EM labelling using an antibody directed to the surface-exposed part of CD9 provided proof that the EV membrane was coated onto the AuNP-BPEI while retaining its orientation (Figure 4B, inserts). To evaluate the functional effect of EV membrane coating, we compared the uptake of AuNP-BPEI and [AuNP-BPEI]^EV^ by J774A1 macrophages using phase contrast microscopy and quantitative Au determination via ICP-MS. This revealed a significant decrease in [AuNP-BPEI]^EV^ uptake, demonstrating successful functionalization of AuNP-BPEI by EV membranes (Figure 4C).

## 4. Discussion

EV are increasingly regarded as suitable therapeutic delivery vehicles, notably in cancer. In this study, we successfully used cancer cell-derived EV membranes to camouflage NP, a first step towards EV-based functionalization of therapeutic nanoparticles.

To evaluate the impact of membrane surface proteins on cancer EV behavior, we compared the uptake of native EV versus proteinase K-treated EV. We confirmed that EV membrane surface proteins play a role in their interaction with cells [18,19,20,33]. Interestingly, we found that intact EV surface proteins resulted in lower uptake by macrophages, while increasing autologous uptake. This is consistent with the notion that EV can evade nonspecific clearance by the immune system and have the potential to be targeted to specific cell types, most probably through homotypic interaction between cell–cell adhesion molecules. Overall, measured autologous uptake of exogenously added 4T1 EV was low. This could be because continuous secretion of EV by 4T1 cells is taking place during the incubation period with exogenously added EV, likely resulting in competition and less uptake of the exogenous EV.

Charoenviriyakul and colleagues used a similar approach with proteinase K to investigate the role of B16BL6 melanoma EV membrane proteins in their pharmacokinetics [33]. While the results in that publication did confirm the targeting aspect of EV membranes, one conflicting observation is that degradation of EV membrane proteins did not affect uptake by macrophages in their case. Besides variations in the model system, an important technical difference between both studies is the type of EV separation method. Our set-up involved density gradient ultracentrifugation to separate EV, as opposed to differential ultracentrifugation. The latter technique typically results in a more crude EV preparation [28]. The presence of these contaminating proteins can influence EV uptake [34]. The fact that the proteinase K concentration necessary to shave off EV membrane proteins is 25 times higher in their case (50 µg mL^−1^ versus 2 µg mL^−1^), further suggests that contaminating proteins were present that compete with EV proteins for the proteinase K enzyme. In our hands, slightly higher concentrations of proteinase K (starting at 3 µg mL^−1^) resulted in destabilization of the EV membrane and degradation of EV contents (Figure S6). Of note, PK-treated EV could not successfully be used to cloak AuNP, possibly due to their increased susceptibility to the pressure exerted during extrusion.

Despite lower uptake, native EV more potently stimulate macrophages to secrete the chemokines MIP-1a and MIP-1b, which are both involved in leukocyte activation after macrophage stimulation. This observation can indicate a dual role for EV membrane proteins. Some proteins can protect EV from nonspecific association with cells, while other membrane proteins result in stronger signaling when EV do end up in macrophages. This might for example be due to a more efficient escape from the phagosome. An alternative explanation is the presence of different subpopulations of 4T1 EV in our preparation. Membrane proteins on some EV might result in macrophage stimulation, while others have membrane proteins that result in avoiding macrophage uptake. We found that while PK-EV are less phagocytosed, macrophages still show phagocytic activity towards native EV (89% uptake of PK-EV versus 64% uptake of native EV after 16 h incubation, Figure S1). Indeed, it has been observed that certain cancer cell-derived EV can readily be taken up by macrophages, with either tumor supporting or tumor suppressing effects [35,36,37].

In this study, the feasibility of extrusion to coat NP with intact EV membranes was confirmed via (immuno) electron microscopy. AuNP were used as proof-of-concept, as they are emerging as promising agents for cancer therapy, not only as drug carriers but also as radiosensitizers or contrast agents [38,39]. BPEI coating of AuNP was necessary for optimal association with EV membranes (Figure S4). Even though this was not addressed in the current study, it is likely that other types of NP, such as PLGA, can be functionalized in a similar fashion after initial coating with a BPEI layer. Our initial experiments showed a role for EV membrane proteins in evading interaction with macrophages, and functionalized AuNP were indeed phagocytosed significantly less as quantified via ICP-MS. The effect of the EV membrane coating, a 13% decrease, was however moderate. This could be due to relatively high (above 60%) uptake of 4T1 EV by J774A1 macrophages, an effect that might be cell-type specific or dependent on EV subtypes. Although we showed that functionalization of NP with cancer cell EV membranes via extrusion is feasible, follow-up studies must evaluate whether this approach will result in better targeting towards tumors and or metastases, and search for optimal EV source cells.

We found that a notable disadvantage of the extrusion approach is its limited efficiency of 3–5%, which is mostly due to AuNP adhering to the polycarbonate membrane. This limited our options for showing a more thorough functional characterization. For each extrusion, 3E10 AuNP-BPEI were combined with E11 4T1 EV which, after washing, resulted in approximately E09 [AuNP-BPEI]^EV^. Unfortunately, the efficiency of mechanical extrusion has not been touched upon in previous papers using the technique for nanoparticle coating [5,9,10,40,41,42]. Another limiting factor is low throughput, as the set-up only allows extruding a total volume of 1 mL at a time. These two factors need optimization before upscaling of the process for extensive in vivo studies and therapeutic purposes in patients. Besides membrane extrusion, other methods for coating nanoparticles with EV membranes have been published. Passive incubation of NP with cells can result in endocytosis and, after passing through the endocytic pathway, release of the NP inside EV, although only for <1% of the particles [43]. Electroporation has been used to actively load EV with chemotherapeutics, but it is unsuitable for larger NP [44,45]. A promising and more controlled approach might be to first isolate EV membrane sheets and subsequently seal them around nanoparticles, as has been successful for leukocyte membrane NP functionalization [7]. Ideally however, functionalizing NP should evolve from a top-down to a bottom-up approach, by designing ‘perfect’ EV biomimetics with desired immune-evading and targeting characteristics [46]. Our understanding of EV biology and interaction with cells is however still limited. Moreover, engineering these synthetic EV with precise control over the presence and relative abundance of a multitude of membrane proteins remains a challenging task.

## 5. Conclusions

This report provides proof of concept for the active cloaking of intact EV membranes on NP. We validated the utility of our approach by showing a role for cancer EV membrane proteins in promoting homotypic interactions with cancer cells, while reducing heterotypic interactions with macrophages. Indeed, EV membrane functionalization reduced NP uptake by macrophages. Our data incentivize more research to leverage EV membrane biomimicking as a unique drug delivery approach in the near future.

## Supplementary Materials

The following are available online at https://www.mdpi.com/2073-4409/9/8/1797/s1, Figure S1: Effect of increasing concentrations of Proteinase K on the stability of intraluminal markers Alix and Flotillin-1, Figure S2: Visible PKH67 dye micelles in fractions 12–13 after bottom-up iodixanol density gradient centrifugation, Figure S3: Concentration of indicated cytokines and chemokines in supernatant of J774A1 macrophages after 16 h stimulation with control medium versus medium containing EV or PK-EV, Figure S4: Representative NTA size distribution and CD9 immunoelectron image of AuNP-BPEI mixed with 4T1 EV, Figure S5: NTA size distributions of AuNP-BPEI and [AuNP-BPEI]^EV^ suspended in water or 10% FBS-containing DMEM, Figure S6: Representative flow cytometry scatter plots of J774A1 cells after 16 h incubation with PKH67 membrane-labelled EV or PK-EV.

## References

[1] Wicki A., Witzigmann D., Balasubramanian V., Huwyler J. (2015). Nanomedicine in cancer therapy: Challenges, opportunities, and clinical applications. J. Control. Release.

[2] Matsumura Y., Maeda H. (1986). A new concept for macromolecular therapeutics in cancer chemotherapy: Mechanism of tumoritropic accumulation of proteins and the antitumor agent smancs. Cancer Res..

[3] Knop K., Hoogenboom R., Fischer D., Schubert U.S. (2010). Poly(ethylene glycol) in drug delivery: Pros and cons as well as potential alternatives. Angew. Chem. Int. Ed. Engl..

[4] Danhier F. (2016). To exploit the tumor microenvironment: Since the EPR effect fails in the clinic, what is the future of nanomedicine?. J. Control. Release.

[5] Hu C.-M.J., Zhang L., Aryal S., Cheung C., Fang R.H., Zhang L. (2011). Erythrocyte membrane-camouflaged polymeric nanoparticles as a biomimetic delivery platform. Proc. Natl. Acad. Sci. USA.

[6] Ai X., Hu M., Wang Z., Zhang W., Li J., Yang H., Lin J., Xing B. (2018). Recent Advances of Membrane-Cloaked Nanoplatforms for Biomedical Applications. Bioconjug. Chem..

[7] Parodi A., Quattrocchi N., Van de Ven A.L., Chiappini C., Evangelopoulos M., Martinez J.O., Brown B.S., Khaled S.Z., Yazdi I.K., Enzo M.V. (2013). Synthetic nanoparticles functionalized with biomimetic leukocyte membranes possess cell-like functions. Nat. Nanotechnol..

[8] Palomba R., Parodi A., Evangelopoulos M., Acciardo S., Corbo C., De Rosa E., Yazdi I.K., Scaria S., Molinaro R., Furman N.E.T. (2016). Biomimetic carriers mimicking leukocyte plasma membrane to increase tumor vasculature permeability. Sci. Rep..

[9] Aryal S., Hu C.-M.J., Fang R.H., Dehaini D., Carpenter C., Zhang D.-E., Zhang L. (2013). Erythrocyte membrane-cloaked polymeric nanoparticles for controlled drug loading and release. Nanomedicine.

[10] Gao W., Hu C.-M.J., Fang R.H., Luk B.T., Su J., Zhang L. (2013). Surface functionalization of gold nanoparticles with red blood cell membranes. Adv. Mater. Weinh..

[11] Ye H., Wang K., Lu Q., Zhao J., Wang M., Kan Q., Zhang H., Wang Y., He Z., Sun J. (2020). Nanosponges of circulating tumor-derived exosomes for breast cancer metastasis inhibition. Biomaterials.

[12] De Wever O., Hendrix A. (2019). A supporting ecosystem to mature extracellular vesicles into clinical application. EMBO J..

[13] Yáñez-Mó M., Siljander P.R.-M., Andreu Z., Zavec A.B., Borràs F.E., Buzas E.I., Buzas K., Casal E., Cappello F., Carvalho J. (2015). Biological properties of extracellular vesicles and their physiological functions. J. Extracell. Vesicles.

[14] Van Niel G., D’Angelo G., Raposo G. (2018). Shedding light on the cell biology of extracellular vesicles. Nat. Rev. Mol. Cell Biol..

[15] Zomer A., Maynard C., Verweij F.J., Kamermans A., Schäfer R., Beerling E., Schiffelers R.M., De Wit E., Berenguer J., Ellenbroek S.I.J. (2015). In Vivo imaging reveals extracellular vesicle-mediated phenocopying of metastatic behavior. Cell.

[16] Armstrong J.P.K., Holme M.N., Stevens M.M. (2017). Re-Engineering Extracellular Vesicles as Smart Nanoscale Therapeutics. ACS Nano.

[17] Fais S., O’Driscoll L., Borras F.E., Buzas E., Camussi G., Cappello F., Carvalho J., Cordeiro da Silva A., Del Portillo H., El Andaloussi S. (2016). Evidence-Based Clinical Use of Nanoscale Extracellular Vesicles in Nanomedicine. ACS Nano.

[18] Kaur S., Elkahloun A.G., Singh S.P., Arakelyan A., Roberts D.D. (2018). A function-blocking CD47 antibody modulates extracellular vesicle-mediated intercellular signaling between breast carcinoma cells and endothelial cells. J. Cell Commun. Signal..

[19] Kamerkar S., LeBleu V.S., Sugimoto H., Yang S., Ruivo C.F., Melo S.A., Lee J.J., Kalluri R. (2017). Exosomes facilitate therapeutic targeting of oncogenic KRAS in pancreatic cancer. Nature.

[20] Hoshino A., Costa-Silva B., Shen T.-L., Rodrigues G., Hashimoto A., Tesic Mark M., Molina H., Kohsaka S., Di Giannatale A., Ceder S. (2015). Tumour exosome integrins determine organotropic metastasis. Nature.

[21] Escudier B., Dorval T., Chaput N., André F., Caby M.-P., Novault S., Flament C., Leboulaire C., Borg C., Amigorena S. (2005). Vaccination of metastatic melanoma patients with autologous dendritic cell (DC) derived-exosomes: Results of thefirst phase I clinical trial. J. Transl. Med..

[22] Yong T., Zhang X., Bie N., Zhang H., Zhang X., Li F., Hakeem A., Hu J., Gan L., Santos H.A. (2019). Tumor exosome-based nanoparticles are efficient drug carriers for chemotherapy. Nat. Commun..

[23] Rivoltini L., Chiodoni C., Squarcina P., Tortoreto M., Villa A., Vergani B., Bürdek M., Botti L., Arioli I., Cova A. (2016). TNF-Related Apoptosis-Inducing Ligand (TRAIL)-Armed Exosomes Deliver Proapoptotic Signals to Tumor Site. Clin. Cancer Res..

[24] Ciullo A., Biemmi V., Milano G., Bolis S., Cervio E., Fertig E.T., Gherghiceanu M., Moccetti T., Camici G.G., Vassalli G. (2019). Exosomal Expression of CXCR4 Targets Cardioprotective Vesicles to Myocardial Infarction and Improves Outcome after Systemic Administration. Int. J. Mol. Sci..

[25] García-Manrique P., Gutiérrez G., Blanco-López M.C. (2018). Fully Artificial Exosomes: Towards New Theranostic Biomaterials. Trends Biotechnol..

[26] Minardi S., Shah S., Luo X. (2018). Biomimetic nanoparticles for transplantation tolerance. Curr. Opin. Organ. Transpl..

[27] Quah B.J.C., O’Neill H.C. (2007). Mycoplasma contaminants present in exosome preparations induce polyclonal B cell responses. J. Leukoc. Biol..

[28] Deun J.V., Mestdagh P., Sormunen R., Cocquyt V., Vermaelen K., Vandesompele J., Bracke M., Wever O.D., Hendrix A. (2014). The impact of disparate isolation methods for extracellular vesicles on downstream RNA profiling. J. Extracell. Vesicles.

[29] Vergauwen G., Dhondt B., Van Deun J., De Smedt E., Berx G., Timmerman E., Gevaert K., Miinalainen I., Cocquyt V., Braems G. (2017). Confounding factors of ultrafiltration and protein analysis in extracellular vesicle research. Sci. Rep..

[30] Cox J., Mann M. (2008). MaxQuant enables high peptide identification rates, individualized p.p.b.-range mass accuracies and proteome-wide protein quantification. Nat. Biotechnol..

[31] Van Deun J., Mestdagh P., Agostinis P., Akay Ö., Anand S., Anckaert J., Martinez Z.A., Baetens T., Beghein E., EV-TRACK Consortium (2017). EV-TRACK: Transparent reporting and centralizing knowledge in extracellular vesicle research. Nat. Methods.

[32] Thul P.J., Lindskog C. (2018). The human protein atlas: A spatial map of the human proteome. Protein Sci..

[33] Charoenviriyakul C., Takahashi Y., Morishita M., Nishikawa M., Takakura Y. (2018). Role of Extracellular Vesicle Surface Proteins in the Pharmacokinetics of Extracellular Vesicles. Mol. Pharm..

[34] Menard J.A., Cerezo-Magaña M., Belting M. (2018). Functional role of extracellular vesicles and lipoproteins in the tumour microenvironment. Philos. Trans. R. Soc. Lond. B Biol. Sci..

[35] Pucci F., Garris C., Lai C.P., Newton A., Pfirschke C., Engblom C., Alvarez D., Sprachman M., Evavold C., Magnuson A. (2016). SCS macrophages suppress melanoma by restricting tumor-derived vesicle-B cell interactions. Science.

[36] Cooks T., Pateras I.S., Jenkins L.M., Patel K.M., Robles A.I., Morris J., Forshew T., Appella E., Gorgoulis V.G., Harris C.C. (2018). Mutant p53 cancers reprogram macrophages to tumor supporting macrophages via exosomal miR-1246. Nat. Commun..

[37] Piao Y.J., Kim H.S., Hwang E.H., Woo J., Zhang M., Moon W.K. (2018). Breast cancer cell-derived exosomes and macrophage polarization are associated with lymph node metastasis. Oncotarget.

[38] Ruan S., Yuan M., Zhang L., Hu G., Chen J., Cun X., Zhang Q., Yang Y., He Q., Gao H. (2015). Tumor microenvironment sensitive doxorubicin delivery and release to glioma using angiopep-2 decorated gold nanoparticles. Biomaterials.

[39] Jain S., Hirst D.G., O’Sullivan J.M. (2012). Gold nanoparticles as novel agents for cancer therapy. Br. J. Radiol..

[40] Fu Q., Lv P., Chen Z., Ni D., Zhang L., Yue H., Yue Z., Wei W., Ma G. (2015). Programmed co-delivery of paclitaxel and doxorubicin boosted by camouflaging with erythrocyte membrane. Nanoscale.

[41] Fang R.H., Hu C.-M.J., Luk B.T., Gao W., Copp J.A., Tai Y., O’Connor D.E., Zhang L. (2014). Cancer cell membrane-coated nanoparticles for anticancer vaccination and drug delivery. Nano Lett..

[42] Khongkow M., Yata T., Boonrungsiman S., Ruktanonchai U.R., Graham D., Namdee K. (2019). Surface modification of gold nanoparticles with neuron-targeted exosome for enhanced blood-brain barrier penetration. Sci. Rep..

[43] Alhasan A.H., Patel P.C., Choi C.H.J., Mirkin C.A. (2014). Exosome encased spherical nucleic acid gold nanoparticle conjugates as potent microRNA regulation agents. Small.

[44] Tian Y., Li S., Song J., Ji T., Zhu M., Anderson G.J., Wei J., Nie G. (2014). A doxorubicin delivery platform using engineered natural membrane vesicle exosomes for targeted tumor therapy. Biomaterials.

[45] Saulis G., Saulė R. (2012). Size of the pores created by an electric pulse: Microsecond vs. millisecond pulses. Biochim. Biophys. Acta.

[46] Kooijmans S.A.A., Vader P., Van Dommelen S.M., Van Solinge W.W., Schiffelers R.M. (2012). Exosome mimetics: A novel class of drug delivery systems. Int. J. Nanomed..

