# Supplementary material for: Feasibility of Mechanical Extrusion to Coat Nanoparticles with Extracellular Vesicle Membranes
Source: Cells. 2020 Jul 29;9(8):1797. doi: 10.3390/cells9081797 (PMC7464356; doi:10.3390/cells9081797)
Supplement: Supplementary file 1 [file cells-09-01797-s001.pdf]

cells

MDPI

Supplementary Information

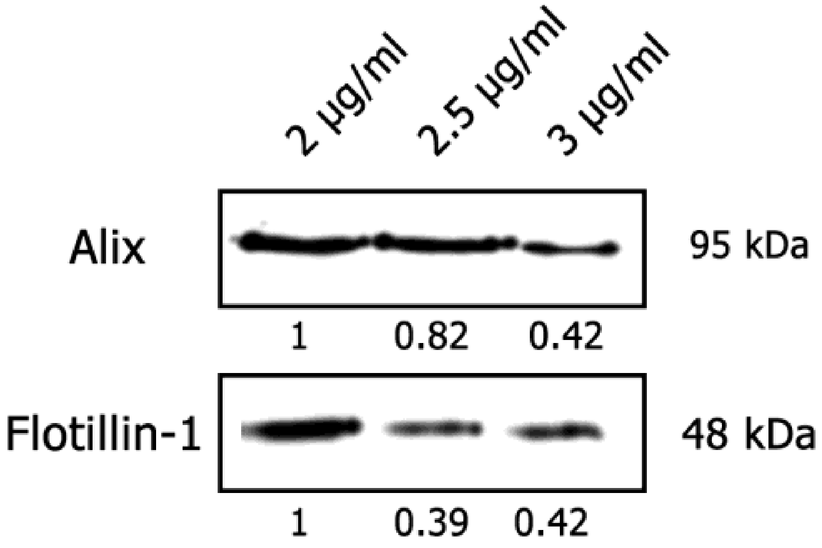

Figure S1. Effect of increasing concentrations of Proteinase K on the stability of intraluminal markers Alix and Flotillin-1. Equal particle numbers were loaded. Band intensities were quantified using ImageJ.

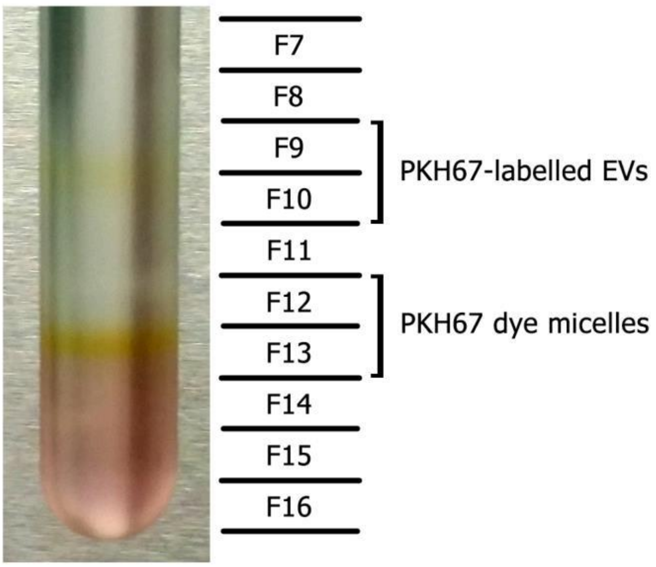

Figure S2. Visible PKH67 dye micelles in fractions 12-13 after bottom-up iodixanol density gradient centrifugation. PKH67 membrane-labelled EV are also observed in fractions 9–10.

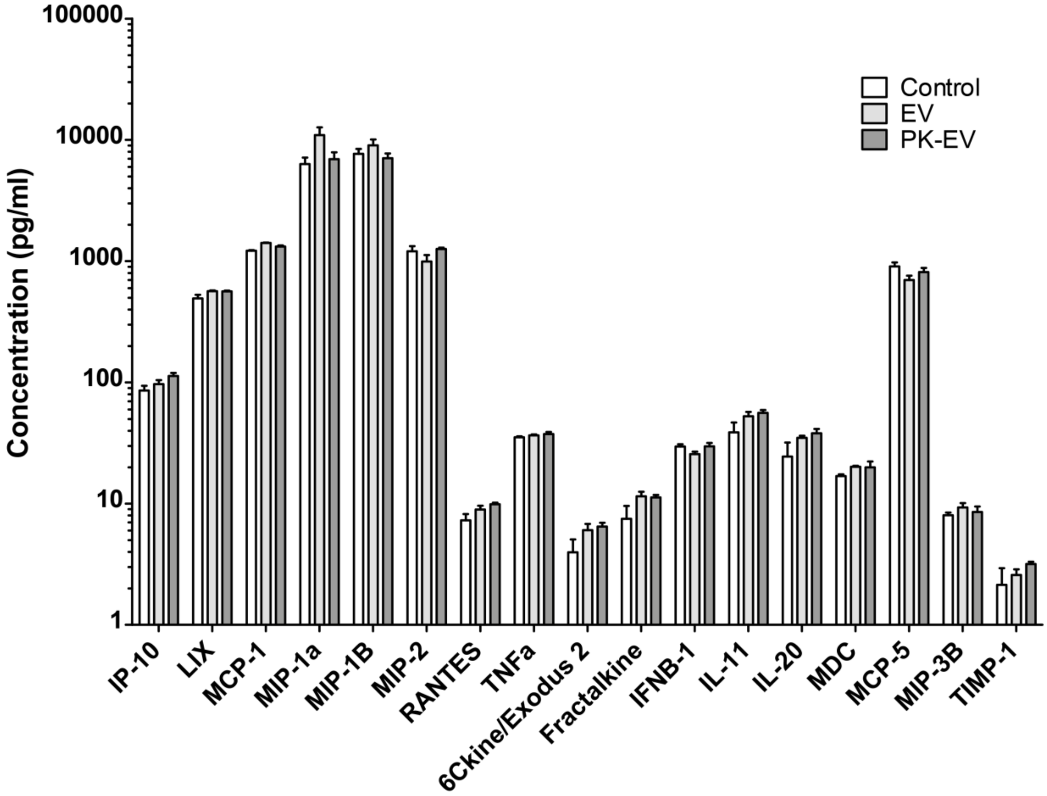

Figure S3. Concentration of indicated cytokines and chemokines in supernatant of J774A1 41 macrophages after 16h stimulation with control medium versus medium containing EV or 42 PK-EV. Mean concentrations are shown with standard error.

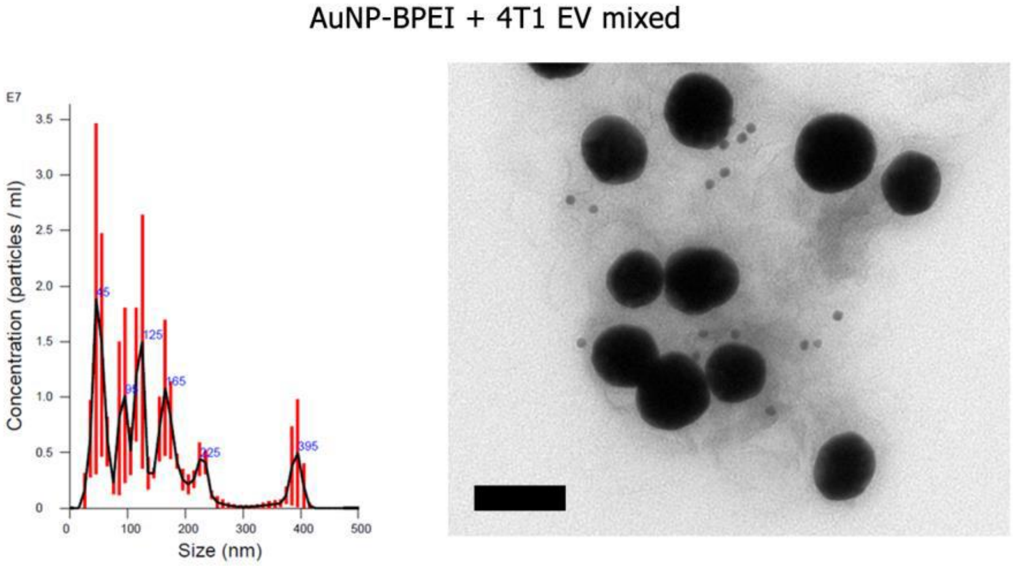

Figure S4. Representative NTA size distribution and CD9 immunoelectron image of 46 AuNP-BPEI mixed with 4T1 EV. Scale bar: 100 nm.

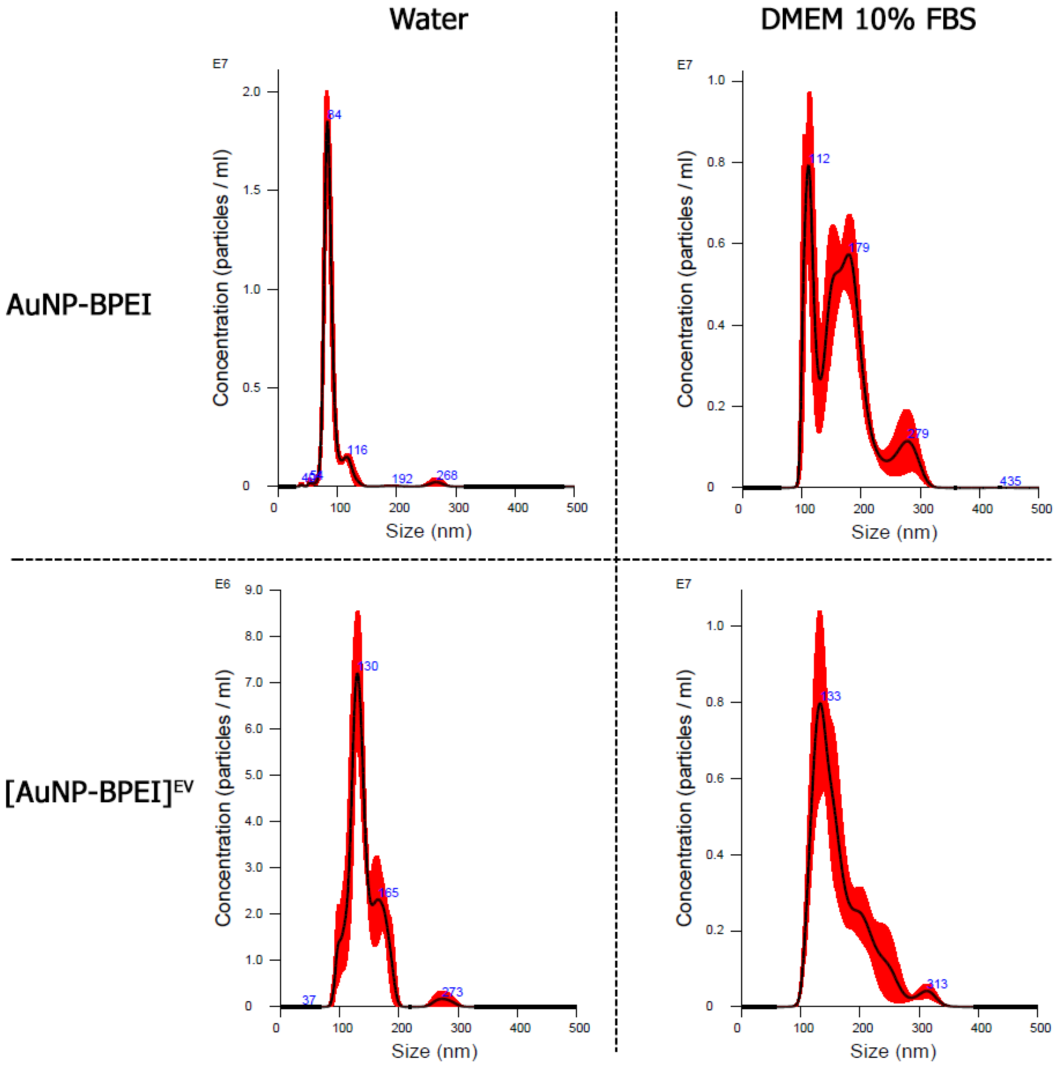

Figure S5. NTA size distributions of AuNP-BPEI and [AuNP-BPEI]EV suspended in water 51 or 10% FBS-containing DMEM.

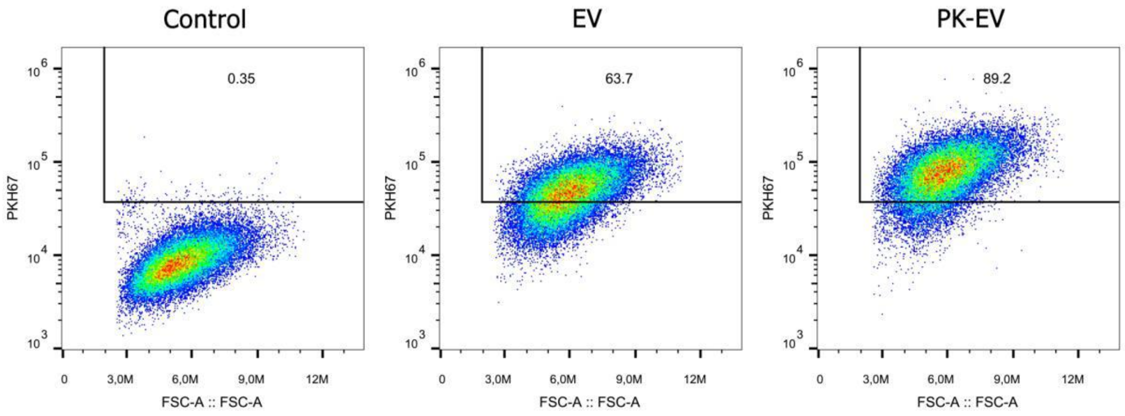

Figure S6. Representative flow cytometry scatter plots of J774A1 cells after 16h incubation 55 with PKH67 membrane-labelled EV or PK-EV. Gating and percentage of PKH67 positive 56 cells are indicated.
